# A Customized Light Sheet Microscope to Measure Spatio-Temporal Protein Dynamics in Small Model Organisms

**DOI:** 10.1371/journal.pone.0127869

**Published:** 2015-05-22

**Authors:** Matthias Rieckher, Ilias Kyparissidis-Kokkinidis, Athanasios Zacharopoulos, Georgios Kourmoulakis, Nektarios Tavernarakis, Jorge Ripoll, Giannis Zacharakis

**Affiliations:** 1 Institute of Molecular Biology and Biotechnology, Foundation for Research and Technology-Hellas, Heraklion, Crete, Greece; 2 Institute of Electronic Structure and Laser, Foundation for Research and Technology-Hellas, Heraklion, Crete, Greece; 3 Department of Bioengineering and Aerospace Engineering, Universidad Carlos III de Madrid, Madrid, Spain; 4 Experimental Medicine and Surgery Unit, Instituto de Investigación Sanitaria del Hospital Gregorio Marañón, Madrid, Spain; 5 Department of Basic Sciences, Faculty of Medicine, University of Crete, Heraklion, Crete, Greece; Tufts University, UNITED STATES

## Abstract

We describe a customizable and cost-effective light sheet microscopy (LSM) platform for rapid three-dimensional imaging of protein dynamics in small model organisms. The system is designed for high acquisition speeds and enables extended time-lapse *in vivo* experiments when using fluorescently labeled specimens. We demonstrate the capability of the setup to monitor gene expression and protein localization during ageing and upon starvation stress in longitudinal studies in individual or small groups of adult *Caenorhabditis elegans* nematodes. The system is equipped to readily perform fluorescence recovery after photobleaching (FRAP), which allows monitoring protein recovery and distribution under low photobleaching conditions. Our imaging platform is designed to easily switch between light sheet microscopy and optical projection tomography (OPT) modalities. The setup permits monitoring of spatio-temporal expression and localization of ageing biomarkers of subcellular size and can be conveniently adapted to image a wide range of small model organisms and tissue samples.

## Introduction

Biomedical research increasingly requires *in vivo* imaging of cellular molecular dynamics as they are unfolding over time in the context of the whole organism. The 3 dimensional (3D) visualization of protein localization, distribution and stability within a specimen allows deciphering complex biological phenomena, such as embryonic development, stress response pathways, and ageing.

Specimens of mesoscopic or macroscopic scales are commonly imaged by Mesoscopic Fluorescence Tomography (MFT) and Fluorescence Mediated Tomography (FMT) allowing efficient 3D depiction [1,2]. Commonly applied methodologies for imaging microscopic model organisms, such as *Drosophila melanogaster* or *Caenorhabditis elegans* are based on differential interference contrast (DIC) and confocal and multiphoton microscopy [3]. In recent years, new imaging technologies capable of producing high-resolution volumetric data sets in the microscopic scale were established, including Optical Projection Tomography (OPT) and the direct optical sectioning technique selective-plane illumination microscopy (SPIM) or light sheet fluorescence microscopy (LSFM) [4,5].

OPT has emerged as a powerful tool for 3D visualization of specimens across 1–10mm in size, combining fluorescence and absorption imaging [6–8]. The technique records projections from multiple equidistant angles perpendicular to the rotational axis of the specimen undergoing one full revolution. Standard filtered back projection algorithms of each angle are used for the reconstruction of all slices, producing 3D volumetric data of the specimen. Initially, OPT was mostly used on fixed specimens after optical clearing in order to reduce photon scattering [9].

We recently reported a versatile OPT setup that allows for microscopic fluorescent imaging of live *C*. *elegans*, with single-cell resolution [10]. However, OPT imaging requires illumination of the whole sample resulting in increased photobleaching of fluorescent samples. The requirement of recording full rotations of the sample imposes also technical difficulties, such as residual random movement and drift of the sample during the course of observation, which have to be corrected by specific algorithms [11,12].

The advent of SPIM permits direct optical sectioning of developing specimens at high penetration depth. This imaging configuration allows optical sectioning, achieved through side-on illumination of the sample by a thin (micrometer-thick) laser light sheet, along a separate optical path orthogonal to the detection axis [13]. One significant advantage of SPIM compared to confocal microscopy is that different regions within the focal plane are simultaneously illuminated and thus exposed for a longer time in average, resulting in reduced photobleaching of fluorophores and fast image recording [14]. SPIM performs well in large samples such as zebra fish and *Drosophila* embryos, and developmental processes can be monitored for several days [15,16]. Recently, SPIM and its modified variant “inverted SPIM” (iSPIM) enabled imaging of cell movements in *C*. *elegans* embryos, which is of importance when measuring cell identity lineaging and tissue development [17,18].

In this work we present and describe in detail a customized, cost-effective LSM, which tackles the challenge of 3D visualization of protein dynamics during ageing in *C*. *elegans*. Our versatile imaging platform integrates the capability to rapidly image fluorescently tagged molecules in individuals or groups of adult animals in longitudinal studies over extended periods in time. Furthermore, the presented system enables recording of fluorescent recovery after photobleaching (FRAP) and protein distribution in the context of the whole organism.

## Materials and Methods

### Sample preparation

*C*. *elegans* is maintained using standard methods [19]. The following strains and transgenic animals are used for this study: N2: wild-type Bristol isolate; *Is*[p_*myo-2*_GFP], expressing GFP in body wall muscles; N2;Ex[p_*unc-119*_GFP;pRF4], expressing pan-neuronal GFP; N2;*Ex*[p_*dcap-1*_DCAP-1::dsRED;p_*ife-2*_IFE-2::GFP;pRF4], expressing dsRED tagged DCAP-1 and GFP tagged IFE-2, each under its endogenous promoter, respectively. For imaging, animals are transferred with a hairpin to agarose pads (5% agarose, Biozym, Germany), which are prepared on glass cover slips (18x18mm, Roth, Germany), and immobilized in nanoparticles (Polybead, Polysterene 0.1μm microspheres, Polysciences, PA, USA) [20]. This procedure avoids the use of anesthetics and keeps animals alive for at least 6 hours. Agarose pads loaded with worms are covered with a glass cover slip. After the imaging procedure, the glass cover slip is removed and animals are recovered by applying a drop of M9 buffer, from where they are transferred by a hairpin to *Escherichia coli* (OP50) seeded NGM agar plates and maintained under standard growth conditions until the next time point of imaging.

### The light sheet microscope

**Fig 1:**
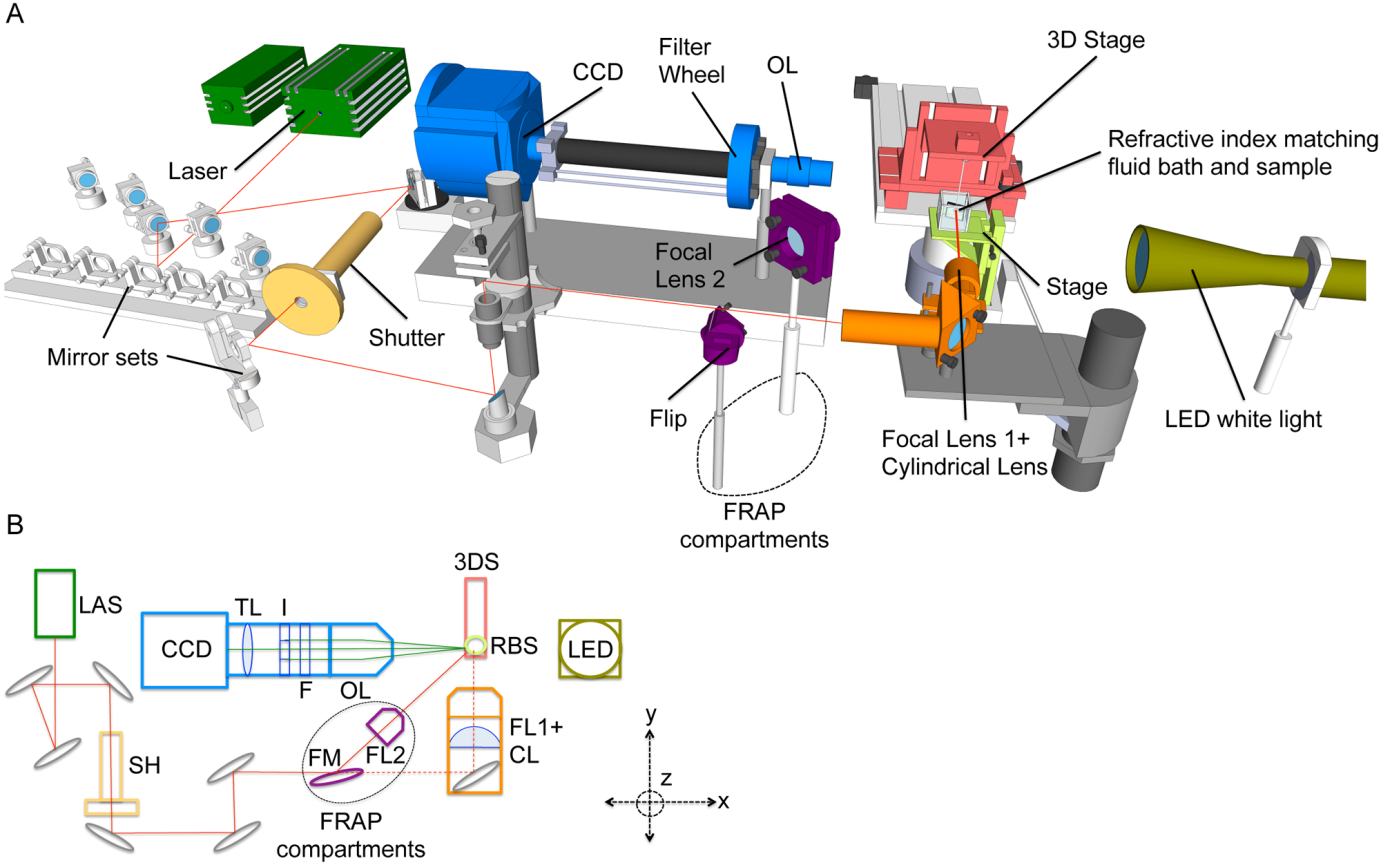
A versatile imaging platform for light sheet microscopy and FRAP. (A) 3D scheme of the LSM and components (see S1 Video for full 3D overview of the setup and compare photographs in S1 Fig). The red line indicates the path of the laser beam. The laser beam is guided by a flexible mirror set to pass through the cylindrical lens, where a laser light sheet is created and focused on the sample, which is immersed in an index matching fluid bath. A 3D stage is used to move the sample along x-, y- and z-axis into position for imaging via the CCD camera with tube lens attachment, which is placed orthogonal to the light sheet. Components for FRAP performance are encircled and consist of a flip mirror and a focal lens to concentrate the beam for fluorescent photobleaching of the sample. A detailed description is provided in Materials and Methods. (B) Basic 2D scheme of the setup showing its essential components. LAS = laser, SH = shutter, FM = flip mirror, CL = cylindrical lens, FL1 = focal lens 1, FL2 = focal lens 2, OS = 3D stage, RBS = Refractive index matching fluid bath and sample, LED = white light LED, CCD = CCD camera, TL = tube lens, I = iris, F = fluorescent filter set, OL = objective lens.

**Fig 2:**
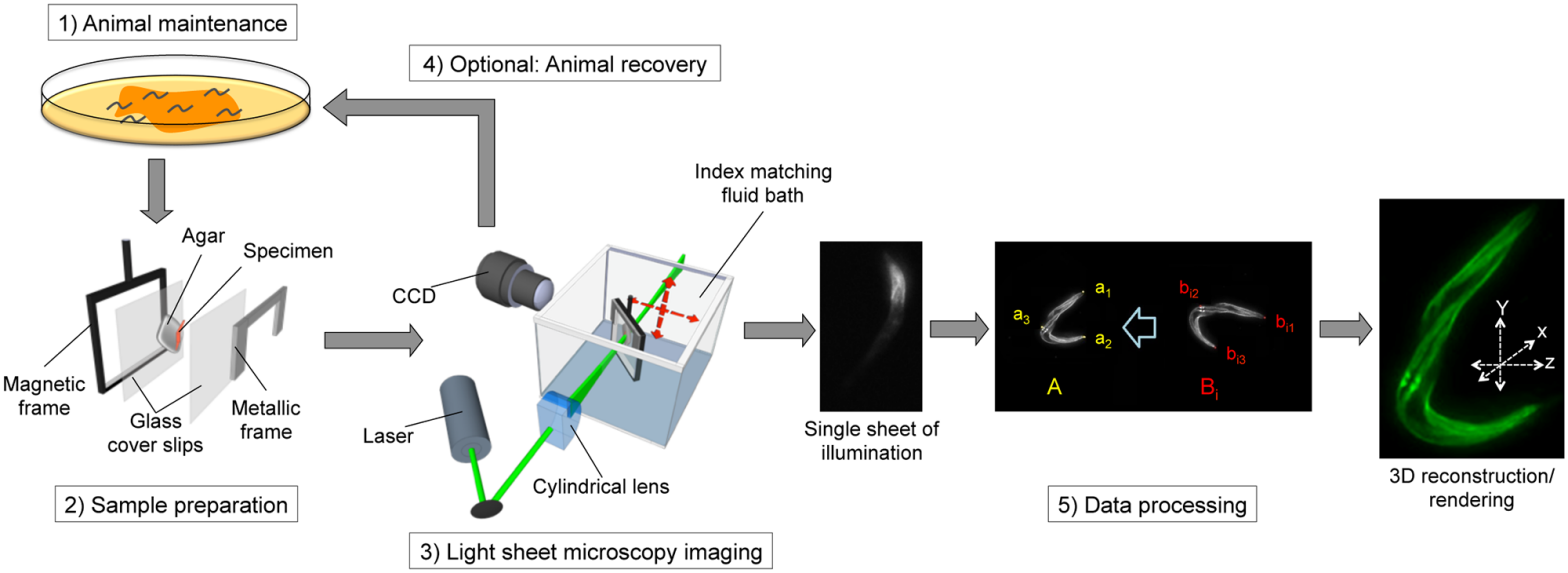
Workflow for light sheet 3D microscopy to measure protein dynamics in *C*. *elegans*. Animals are maintained following standard procedures, collected and immobilized on agar pads, which are immersed in an oil bath. The laser beam for sample illumination passes through a cylindrical lens and forms a light sheet that is directed on the specimen. The recording lens is focused on the illuminated sheet and records stacks of the specimen, which is moved along the x-axis. Recorded data sets consist of image stacks and are combined into a 3D visualization via freeware, such as ImageJ. Anatomic landmarks (b_i1_, b_i2_, b_i3_) are used to map and transform images via registration algorithms so that they can be aligned to a reference image A (see Materials and Methods).

All compartments of the setup, including possible vendors are summarized in S1 Table. The system is highly versatile and can easily be adjusted for specific needs of imaging (see Fig 1A and 1B, S1 Fig and S1 Video). Light sources for sample illumination are lasers of various wavelengths, allowing excitation of most commercially available fluorescent dyes, including 488nm (GFP excitation) and 532nm (dsRED or mCherry excitation). Various adjustable mirrors direct the laser beam through a shutter (Vincent Associates Rochester, NY, USA) and a cylindrical lens (CL; LJ1960L1-A, focal length 20mm, Thorlabs, Germany) to produce the light sheet, which is focused on the sample through an infinity-corrected microscope lens (focal lens, FL1; M Plan Apo 5x, NA = 0.14, Mitutoyo, Kawasaki, Japan). The width of the light sheet is <10 microns, as measured on an irregular glass surface (S1 Fig). A versatile arrangement of adjustable mirror sets allows selective switching between the lasers according to imaging needs. A white light LED is installed for transmission (absorption contrast, brightfield) imaging and supports focusing on the sample. Samples are placed between a custom-made magnetic frame and its metallic counterpart (Fig 2). The sample holder is attached to a 3D stage (OS, 8MT167-100, Standa, Vilnius, Lithuania), which permits movement of the sample along x-, y- and z-axis, and a rotation system, containing a high-resolution rotation stage (8MR180, Standa, Vilnius, Lithuania) with 36000 steps per revolution. The whole sample is immersed into a refractive index matching cell (height 52.5mm, width 55mm, depth 55mm, Hellma Analytics, Germany), which is positioned on a lifting stage and filled with refractive index matching fluid (Baby Oil, n_f_ ≅ 1.47, Nivea, Germany) to minimize internal reflections and refraction of the excitation and emission light. The imaging unit is placed perpendicular to the excitation beam and consists of a lens tube system (InfiniTube, Infinity, Boulder CO, USA) attached to a high-speed motorized filter wheel (F, FW103/M, Thorlabs, Newton, NJ, USA), which can hold up to five 25mm diameter fluorescence filters (531nm/40::FF01-531/40-25 [25mm], Semrock, NY, USA, and 605nm/70::ET605/70M [25mm], Chroma, VT, USA, for this study). We used a 10x infinity corrected microscope objective lens (Mitutoyo, Kawasaki, Japan). The objective lens (OL) has a numerical aperture NA of 0.28, which is reduced to typically around 0.22 by the iris diaphragm. The NA for trans-illumination is <0.035, for fluorescence illumination it is ca. 0.1. Except for the collimation lens, all lenses are corrected for chromatic aberrations. The detection filter and the iris are positioned next to the back focal plane of the objective. Depending on the specimen size, infinity corrected lenses with a range of other magnifications are available. The objective lens is attached to a thermoelectrically cooled, electron multiplying CCD with 1002x1004 pixels (Ixon DV885, ANDOR Technology, Belfast, Northern Ireland). To increase the focal depth of the system, a variable iris (I) is placed behind the objective [21].

### Fluorescence Recovery After Photobleaching (FRAP)

For quenching fluorescence in samples the laser beam of the 488nm (for GFP) or 532nm (for dsRED) lasers is directly targeted on the sample by bringing the adjustable “flip mirror” (FM, FM90 with KMS, Thorlabs, Newton, NJ, USA) in an upright position, which bypasses the lens for light sheet conversion (Fig 1A and 1B). A focal lens (FL2, 10x objective, NA = 0.28, WD = 33.5mm, Mitutoyo) concentrates the light beam on specific areas in the sample. Continuous imaging of the sample controls the reduction of fluorescent intensity in the area of interest to 10–20% of the initial signal (depending on signal intensity and target tissue 5-10min). Since the redirected beam is not perpendicular to the sample cell and laser power is significantly reduced when passing through the oil bath, bleaching is performed in the air. Thereafter, the sample is lowered into the index matching fluid cell and imaged by light sheet microscopy, which requires switching the FM in its down position. Recovery of fluorescence is typically monitored every 30 min for 3 hours. Proper photobleaching conditions (light intensity, duration) were chosen to avoid injury to worms. After completing the imaging session animals were recovered and maintained at standard growth conditions to score for damage induced by the imaging procedure. The animals were observed for obvious behavioral (egg laying, locomotion) and morphological changes for 3 days (data not shown).

### Data acquisition and processing

Data sets are acquired using control software developed by 4D-Nature (4D-Nature Imaging Consulting, S.L., Madrid, Spain) to control (I) specimen positioning and movement through the light sheet along the z-axis, (II) the camera focus, (III) image recording and (IV) the rotation stage. The graphical user interface (GUI) controls all relevant parameters (camera, stage movement, and filters) and allows for convenient design of automatic data acquisition sequences for imaging experiments, including light sheet microscopy, OPT, extended time lapse and video-recorded live imaging. A typical data set for a single light sheet microscopy recording consists of a stack of 80–120 images (1000x1000 pixels each) of the plane of illumination through the specimen taken in 0.007mm steps and stored as one collected file with a size of approximately 200 megabyte.

Image processing starts by creating volumetric images, one for each of the different time-points, by stacking together the sequential images acquired along the depth axis of the experimental setup. Those slices are then corrected for any misalignments or motion during the experimental procedure to create the final result. The images corresponding to different time-steps or multiple viewing angles are sequentially inserted in a rigid and non-rigid registration process. In general, it is sufficient to use rigid-affine models to deal with multispectral images and both rigid and non-rigid models for the images acquired from different viewing angles. The global motion of the specimens between the different images was modelled by an affine transformation, while the local motion and finer misalignments are described by a free-form deformation based on B-splines [22,23]. The alignment between the different image stacks is guided by voxel-based similarity measures based on normalized mutual information and highlighting anatomical landmarks defined on the specimen’s volumes. From each series of samples, we picked one as the target volume, noted A, and for each other sample in the sequence B_1_, B_2_, etc., we defined the transformation that would align B_i_ (i = 1, 2, etc.) to A. When landmarks were used, they were first defined as features of the target image A, for example a_1_, a_2_, a_3_, etc., and then in each of the volumes B_i_ as their corresponding points b_i1_, b_i2_, b_i3_, etc. (see S2 Fig). Those points are used to calculate the transformation needed for alignment, which is then used to map and transform the second image, so that it aligns with the target image, by applying a registration algorithm for rigid and non-rigid transformations. In case that this results in a transformation that after visual inspection is found to be unsatisfactory, more landmarks are being defined and the transformation is re-applied. S2 Fig displays the resulting image when the two wavelengths (green and red) are superimposed before the registration and the aligned images (S2B Fig), using the procedure described.

### Statistical Analysis

ImageJ (http://imagej.nih.gov/ij/) was used to determine average or mean pixel intensities of raw data sets before 3D reconstruction. For statistical analyses we apply the Prism software package (GraphPad Software Inc., San Diego, USA). Mean values are compared using unpaired t tests. The linear regression tool of the Graph Pad Prism is used to generate best-fit lines corresponding to fluorescence recovery rate, which produces best-fit values, goodness of fit, slope and x-/y-intercept results.

## Results and Discussion

Here, we describe a customized, cost-effective and versatile LSM that generates 3-dimensional (3D) data sets of protein dynamics in microscopic specimens. Fig 1A and 1B, and S1 Video display the individual components of the system and their arrangement (for a full list of components see S1 Table). For short- and long-term imaging, nematodes are immobilized in nanoparticles on agar pads (5–10%), which makes the use of anesthetics such as levamisol or sodium azide unnecessary, and allows recovery of the animals to optimal growth conditions after the imaging procedure [20]. The agar pads are sealed with a glass cover slip and placed into a magnetic sample holder, which is attached to the objective stage of the LSM and subsequently immersed in an index matching fluid bath (see Fig 2 and Materials and Methods).

The objective stage moves the sample in all three dimensions and is controlled by custom-made software. The sample is positioned in the trajectory of the light sheet and the imaging system (CCD camera and objective lens) is focused on the plane of illumination in a perpendicular angle. For imaging, the objective stage is programmed to move the sample in equidistant steps along the z-axis through the light sheet. Each step is recorded by the imaging system, producing a dataset of in-focus image stacks, which can be processed into a 3D image by standard freeware (ImageJ, for details on reconstruction procedure see Materials and Methods). Source of illumination for the present study are a white light LED and two laser sources (488nm, excitation of GFP, and 532nm, excitation of dsRED). The system is readily equipped with a wide range of lasers and LEDs of different wavelengths for illumination of the specimen depending on the fluorophore to be excited (see S1 Table). The appropriate fluorescence filters are implemented, which enables sequential imaging of different chromophores within the same specimen. Thus, the system is optimized to image a variety of fluorescent marker proteins, fluorescent dyes for cell- or tissue-specific labeling of an organism and endogenous fluorophores (autofluorescence). The light sheet microscopy setup allows image acquisition at video rate and depending on the intensity of the fluorescent signal, up to 120 stacks can be recorded in less than 2 min.

**Fig 3:**
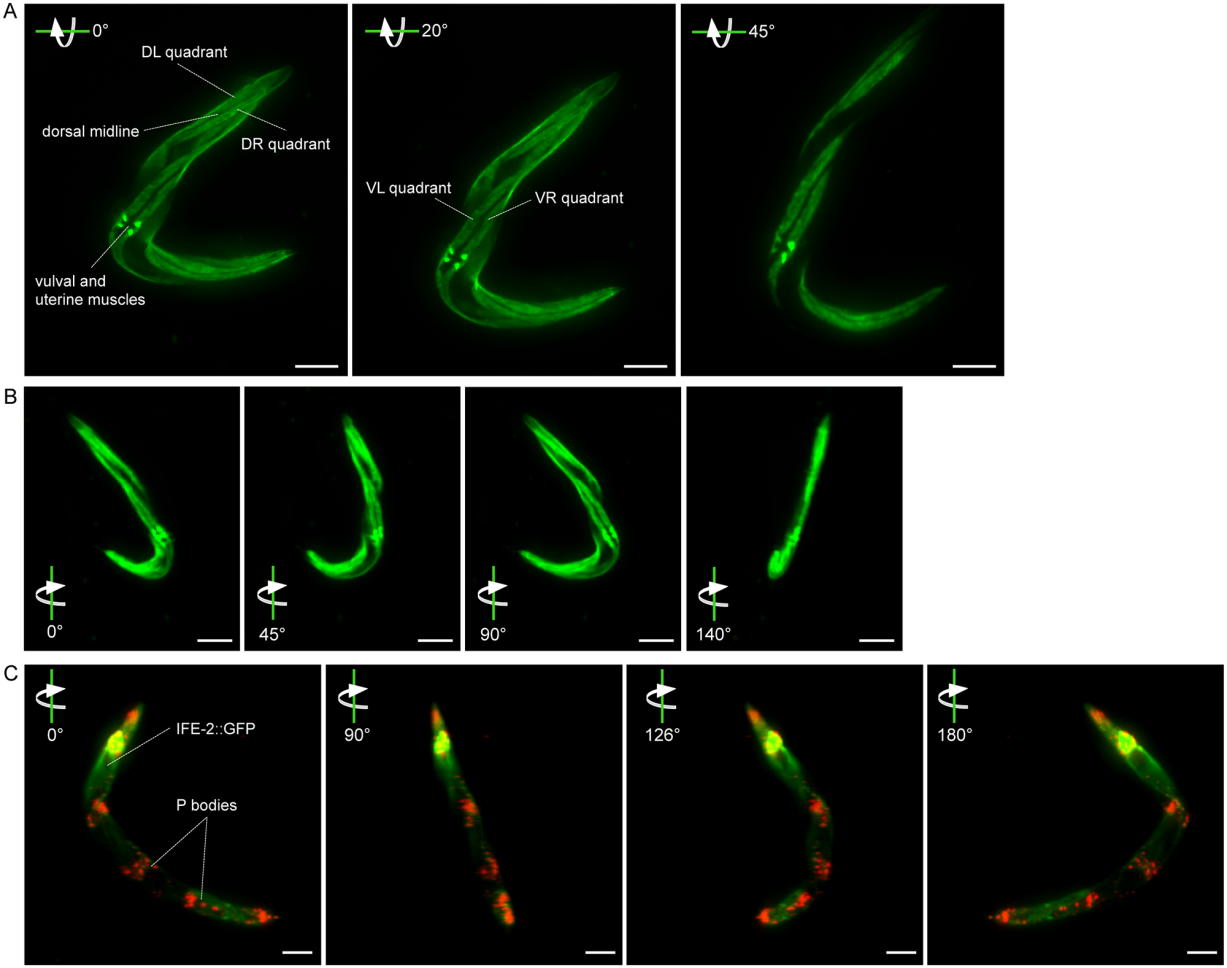
3D visualization of *C*. *elegans* derived from whole animal recording by light sheet microscopy. (A) Representative images of a multiangle acquisition (0°, 20° and 45°) of an animal expressing muscle-specific GFP (p_*myo-3*_GFP). The 3D visualization allows the characterization of body wall muscle-quadrants and vulval muscles. DL = dorsal-left, DR = dorsal-right, VL = ventral-left, DL = dorsal-left. Size bars correspond to 100μm. (B) View from several angles (0°, 45°, 90° and 140°) on a fully reconstructed 3D image of the animal displayed in (A), which is also depicted in S2 Video. Size bars correspond to 100μm. (C) 3D image of transgenic *C*. *elegans* co-expressing the P body reporter p_*dcap-1*_DCAP-1::dsRED and the translation initiation factor eIF4E, p_*ife-2*_IFE-2::GFP. The animal is depicted from four different angles. A full rotation is shown in S3 Video. Size bars correspond to 100μm.

To demonstrate the imaging capacity of our custom-built LSM presented in Fig 1A and 1B we imaged transgenic *C*. *elegans* animals *in vivo*, carrying a green fluorescent protein (GFP) reporter fusion expressed specifically in the body wall musculature of the nematode [24]. The labeled muscle cells are localized in the vulva and the longitudinal bands surrounding the animal body, with one band running in each quadrant (Fig 3A and 3B and S2 Video). The images in Fig 3A are acquisitions from different angles, which were then co-registered and superimposed to create a full 3D reconstruction, which is displayed in Fig 3B and S2 Video.

Further, we monitored transgenic animals co-expressing the GFP tagged translation initiation factor IFE-2, and the dsRED labeled decapping activator DCAP-1, each driven by their endogenous promoter, respectively (p_*ife-2*_IFE-2::GFP; p_*dcap-1*_DCAP-1::dsRED). IFE-2 shows ubiquitous expression in all somatic tissues of *C*. *elegans*, while DCAP-1 localizes into Processing bodies (P bodies), which are subcellular foci that regulate mRNA stability and transport in all tissues of the animals [25,26]. Our 3D recordings verify the broad distribution of the two proteins in the organism (Fig 3C and S3 Video). P bodies are cytoplasmic granules that vary in size from submicron (100-300nm) to several microns in diameter [26,27]. Despite their size of 1 to 3μm, P bodies are clearly visible, showing the capacity of the system for highly sensitive 3D visualization of subcellular structures, within the context of a whole organism. Visualization and tracking of subcellular compartments or regions in *C*. *elegans* including P bodies, lipid droplets, mitochondria, or vesicles like lysosomes or autophagosomes, is essential to study basic mechanisms of cell metabolism.

Fig 3 demonstrates the possibility for volumetric rendering of fluorescently labeled tissues and subcellular structures in the context of a whole organism. Light sheet microscopy is incapable of recording intrinsic or extrinsic absorption, which makes the display of anatomical information less efficient [14]. To overcome this limitation, all tissues of the animal can be labeled with fluorescence and combined with the data sets derived from the chromophore of interest. Data of the ubiquitous expression of IFE-2::GFP, which labels all somatic tissues, merged with recordings of DCAP-1::dsRED allows for spatio-temporal analysis of P body localization in the context of the entire animal. Further, we observe co-localization of the two fluorescent factors in various tissues including the pharynx, vulva, some muscles and the tail region (Fig 3C and S3 Video).

The presented system is capable of optical projection tomography (OPT), a relatively recently developed technology for 3D imaging that we had adopted for monitoring microscopic specimen *in vivo* [4,10]. Therefore, the setup combines the potential for simultaneous fluorescence imaging by light sheet microscopy with absorption-derived anatomical/structural data resulting from OPT.

Furthermore, the spatio-temporal analysis of protein localization within a cell or a whole organism is of central importance to understand biological processes, such as ageing and development. Ageing is a stochastic phenomenon, where animal populations are considered for age-related phenotypes, such as protein dynamics [28]. However, the study of individuals can unravel molecular variability within a population and reveal novel aspects that might be missed when looking at large animal pools [29]. To study the dynamics of the two proteins during aging in individuals we used our LSM to image transgenic animals carrying reporters for IFE-2::GFP and DCAP-1::dsRED in a longitudinal study.

**Fig 4:**
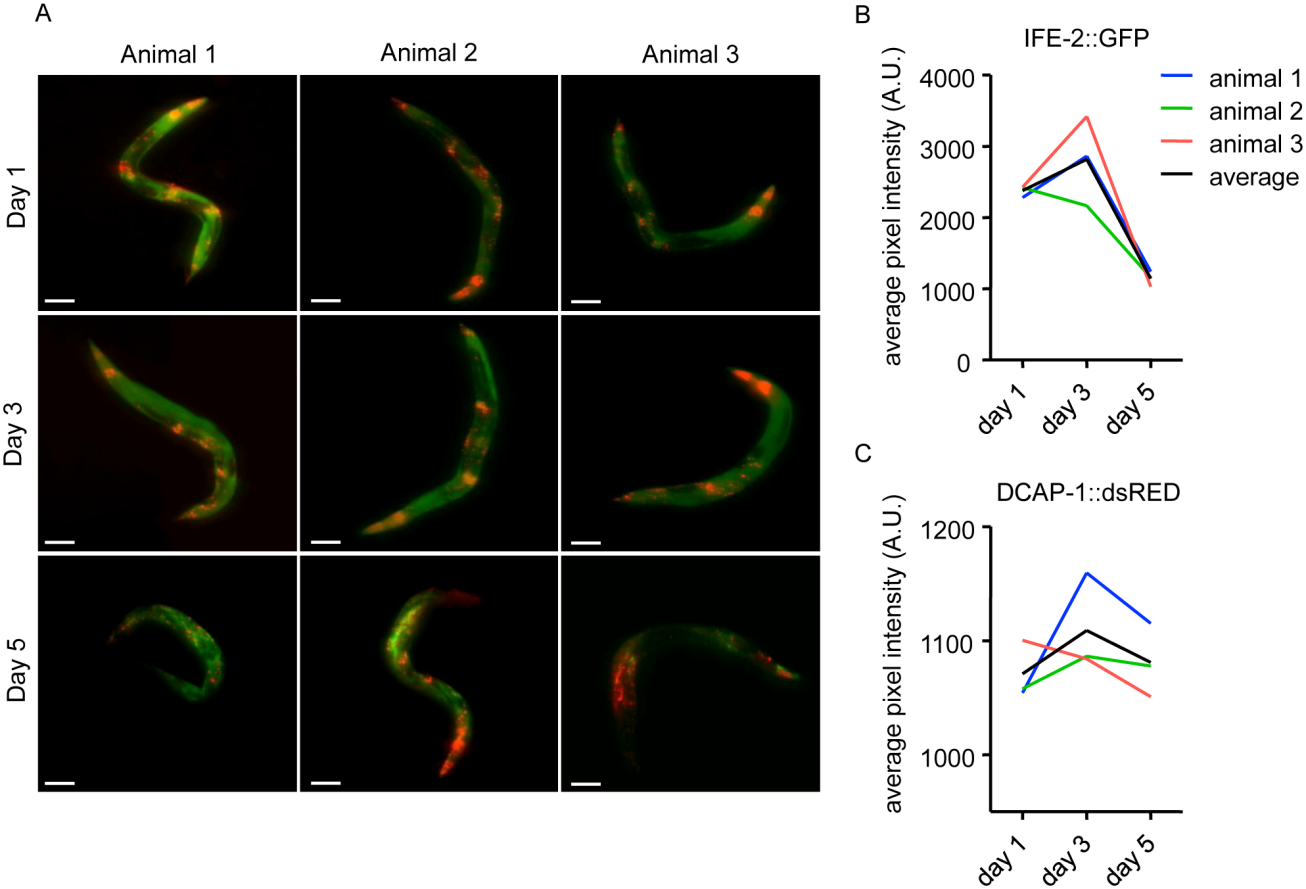
Protein dynamics in a longitudinal ageing study in *C*. *elegans*. (A) Representative images of 3 single animals grown at 25°C at day 1, 3 and 5. Animals co-express p_*ife-2*_IFE-2::GFP and p_*dcap-1*_DCAP-1::dsRED (see S4 Video). Individuals show differences in protein localization during ageing. Size bars correspond to 100μm. (B) IFE-2::GFP and (C) DCAP-1::dsRED fluorescent intensity quantification of the 3 animals in (A).

IFE-2, a homologous isoform of eIF4E, has been shown to modulate *C*. *elegans* life span through its function in protein synthesis and cellular energy metabolism [25]. DCAP-1 activates decapping and degradation of mRNAs and increasingly localizes to P bodies in adult animals [26]. Both factors play a central role in the regulation of mRNA translation [30]. We followed the protein localization within three animals in a longitudinal study at day 1, day 3 and day 5 of adulthood (Fig 4A–4C and S4 Video for animal 3). After each round of imaging, animals were recovered from agar pads and reared under standard growth conditions until the next imaging time point (Fig 2).

Curiously, we observed that protein expression patterns for IFE-2::GFP and DCAP-1::dsRED changed differentially within the individual organisms and between time points (Fig 4A, compare S4 Video for animal 3). From day 1 to day 3, intensity of both fluorescent reporters increased significantly, which was followed by a drop of intensity levels at day 5. Exceptions were animal 2, for IFE-2::GFP, and animal 1, for DCAP-1::dsRED, which showed stepwise decrease of expression levels, respectively (Fig 4B and 4C). The peak of fluorescent intensities at day 3 point to an increased requirement of both factors during the reproductive period of adult *C*. *elegans*. Production of oocytes ceases at day 4 of adulthood, possibly accounting for the decreased expression of IFE-2. Levels of DCAP-1 at day 5 of adulthood do not drop below levels of day 1, indicating a continuous post-reproductive necessity of the protein for cellular mechanisms.

**Fig 5:**
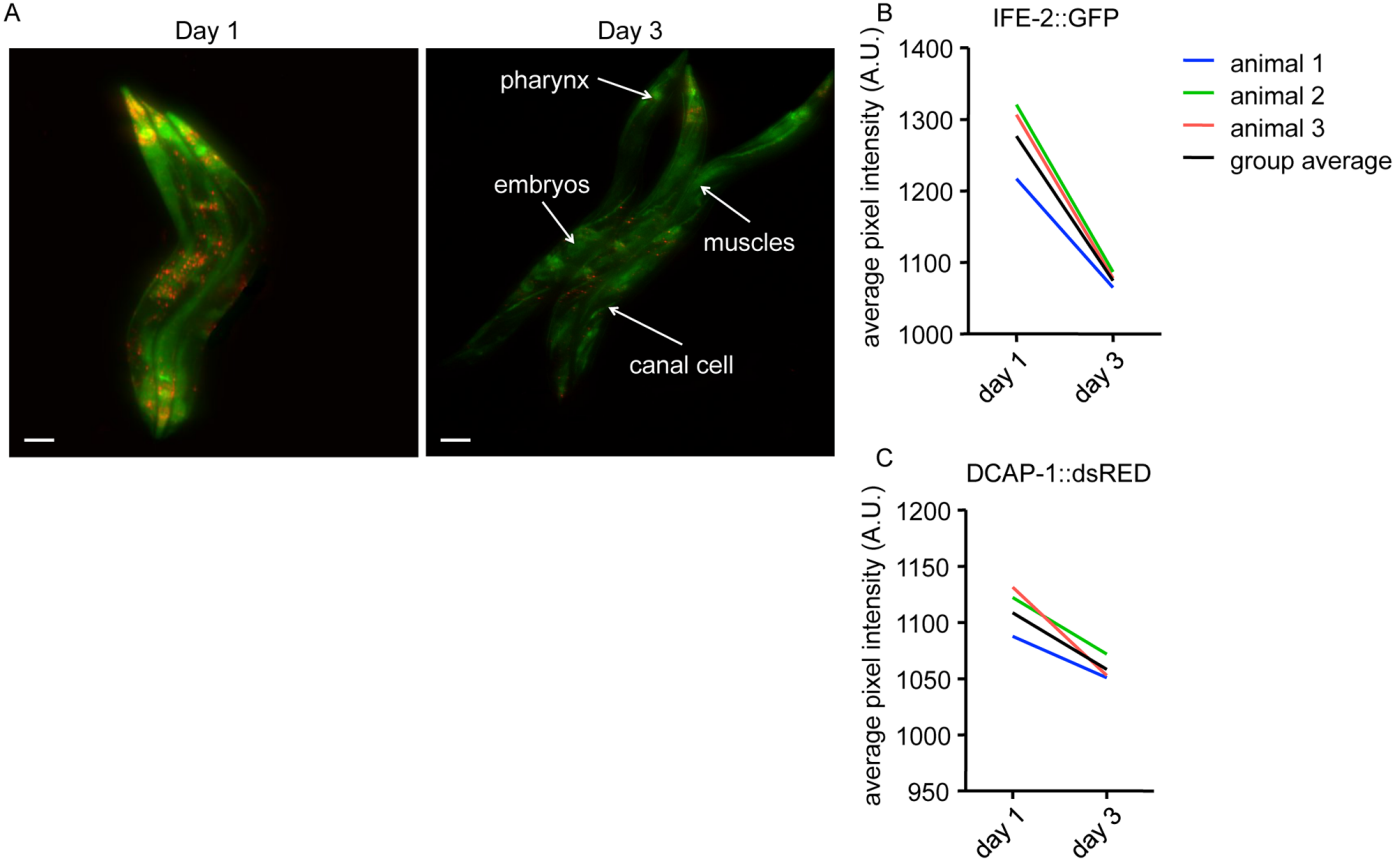
Changes of protein localization upon starvation-induced stress. (A) Representative images of 3 starved animals grown at 25°C at day 1 and 3 in a group. Decrease of fluorescence for both reporters p_*ife-2*_IFE-2::GFP and p_*dcap-1*_DCAP-1::dsRED from day 1 to day 3 are visible, while the signal persists in some tissues, including pharynx, canal cells, muscles and the developing embryos (see S5 Video). Size bars correspond to 100μm. (B) IFE-2::GFP and (C) DCAP-1::dsRED fluorescent intensity quantification of the 3 animals in (A) individually and in the group.

We were curious to see whether the differential expression of IFE-2::GFP and DCAP-1::dsRED can also be observed upon induction of environmental stress. To demonstrate this, we imaged a group of three adult animals grown under optimal condition, and after recovery shifted them to agar plates lacking food. 48 hours later, we reexamined the same group and order of animals. We observed changes in protein localization for both factors. In particular, IFE-2 shows persistent expression in muscles, pharynx and the canal cells, as well as in developing embryos (Fig 5A and S5 Video). Further, we measured a significant but differential decrease for both factors in all three animals (Fig 5B and 5C). Starvation results in a general decrease in activity of cellular metabolic pathways and protein synthesis, which could explain the reduced levels of IFE-2 and DCAP-1, which are factors involved in translation regulation [31]. Together, these results demonstrate that the presented setup is capable of measuring protein dynamics in longitudinal studies during ageing and stress response in *C*. *elegans*.

The proper regulation of protein synthesis and movement is essential for cellular and organismal development and ageing. A wide range of fluorescent reporter fusions expressed in specific cells and tissues is available for *C*. *elegans* (e.g. the *C*. *elegans* Gene Expression Consortium) [32]. Hence, cell- and tissue-specific synthesis and diffusion of *de novo* protein in *C*. *elegans* can be studied by FRAP [33,34]. We designed our LSM to allow for photobleaching of fluorescence-tagged proteins in specific areas of the animals and subsequent 3D microscopy to visualize protein recovery. Fig 1A and 1B show the installation of a “flip mirror” (FM), which is put into an upright position to target the laser beam on the sample for photobleaching. Subsequently, the mirror is reversed to direct the light beam through the focal lens for light sheet imaging (see Materials and Methods).

**Fig 6:**
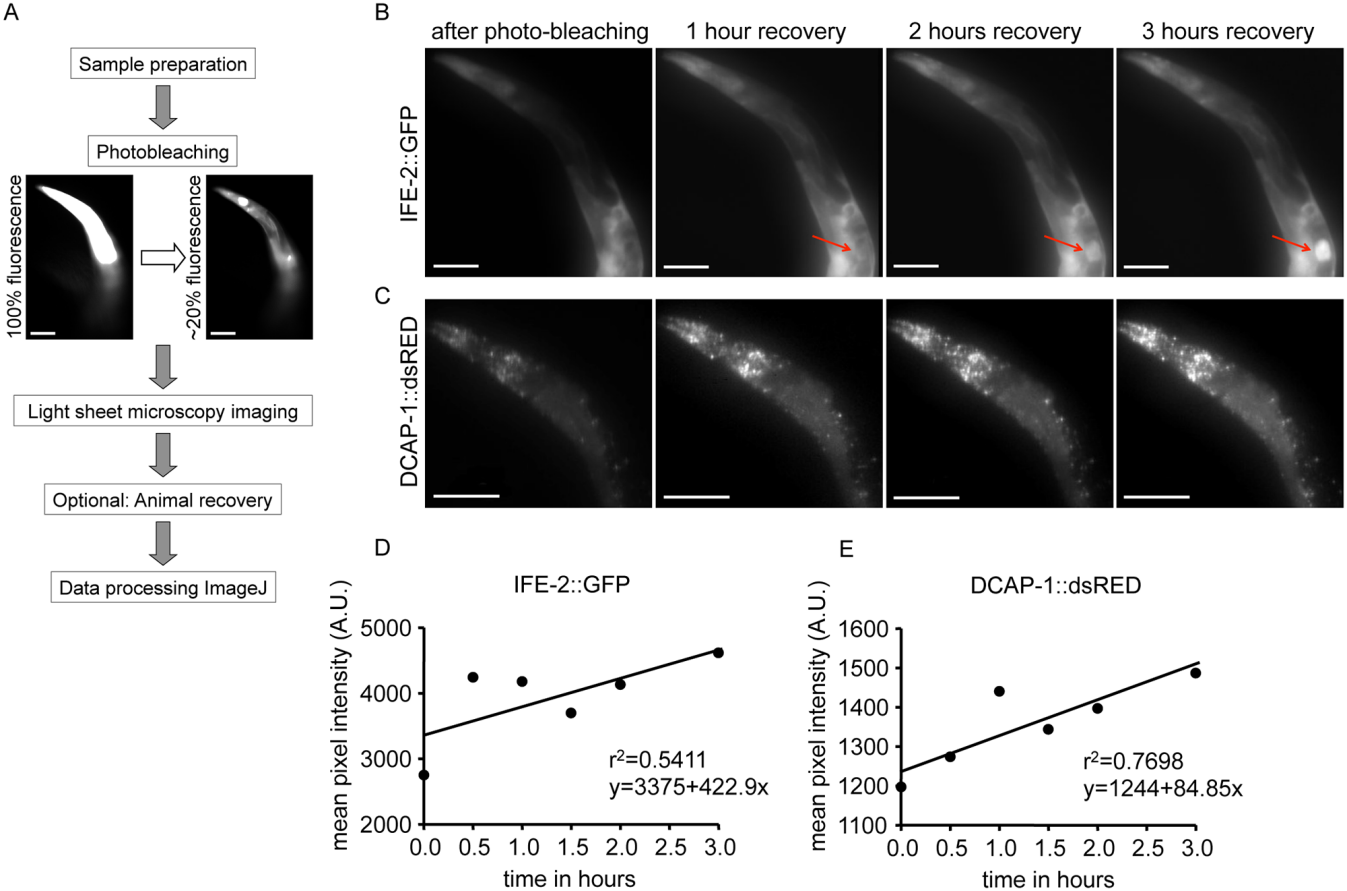
FRAP imaging by low-photobleaching light sheet microscopy. (A) Workflow for FRAP imaging by light sheet microscopy. After monitoring, the animals can be recovered to assess possible damage induced through the procedure. (B) Representative images of recovery at several time points after photobleaching IFE-2::GFP fluorescence in the anterior part of the animal (see S6 Video). The red arrow indicates a developing embryo within the parental gonad. Size bars correspond to 100μm. (C) Imaging of the anterior region after photobleaching and recovery time points of DCAP-1::dsRED fluorescence. The signal also recovers to the subcellular structures of P bodies (see S7 Video). Size bars correspond to 100μm. (D) IFE-2::GFP and (E) DCAP-1::dsRED quantification of recovering fluorescent intensity of the animals in (B) and (C), respectively. The respective equations describing best-fit lines as well as r^2^ values for each graph are shown. The line slopes correspond to the first derivative of fluorescent change within a time unit (df/dt) and determine the recovery rate.

We asked whether our system can detect *de novo* synthesis of IFE-2::GFP and the P body reporter DCAP-1::dsRED, respectively. To answer this question, we depleted the fluorescence signal from the anterior region of the animals to 10–20% of pre-bleaching intensity. Recovery of fluorescence, which indicates new protein synthesis, was then monitored in the anterior part of the animals over the time course of three hours. The collected data sets for each imaging round were analyzed for fluorescent intensity to quantify new protein synthesis and protein diffusion in tissues. Animals were recovered after the imaging procedure and maintained for at least three days (Fig 6A). No physiological damage due to the procedure could be recognized (morphology, behavior in egg-laying and locomotion, data not shown). We observed recovery of both factors, while IFE-2::GFP shows a faster recovery rate compared to DCAP-1::dsRED, which is determined through the line slope of the best-fit line (Fig 6B–6E). Analysis of 3D data sets allows defining differential recovery and distribution of fluorescent signal in specific tissues (S6 Video, IFE-2::GFP, S7 Video, DCAP-1::dsRED, and S8 Video, merge of the signals). Further, we performed FRAP in animals that constitutively express GFP in the neuronal system. Fluorescence was depleted in the anterior part of the animal and we observed signal recovery in a subset of neurons located in the tail region, while neurons outside the area of photobleaching did not show any signal increase (S3A–S3C Fig).

We demonstrate that our customized setup has the capability for performing FRAP experiments in specific tissues and areas within the organism, which allows studies of protein dynamics during development and ageing. Most FRAP studies are performed by laser scanning confocal microscopy. Repetitive scanning of the bleached region of interest to image signal recovery further reduces the signal and might disturb the final readout [34]. In LSM-based imaging, fluorophores are only excited in the illuminated plane, avoiding photobleaching or photoinduced damage in the surrounding regions [35]. Since our approach employs a low photobleaching imaging technique for monitoring *de novo* protein synthesis it might offer higher accuracy by preventing further photobleaching. Further, our setup permits visualization of developmental processes in *C*. *elegans*. Fig 6B shows the maturing of an embryo, indicated by the appearance of fluorescent expression over the time course of 3 hours (red arrows).

In summary, we present a cost-effective LSM, which is easy to assemble and customize, and achieves fast 3D recording of live small specimen. Our setup is highly adaptable and allows for multi-fluorescent imaging with low photobleaching. By application of free software, such as ImageJ, we achieve 4D visualization of tissues, single cells and subcellular structures, like P bodies, in live adult *C*. *elegans*. Thus, the system is specifically suited to image gene expression patterns and protein dynamics over extended periods of time. The protocols for immobilization of individual or small groups of animals using nanoparticles allow longitudinal studies and monitoring of protein localization during the course of development and ageing.

Further, our setup is readily equipped to perform FRAP assays in specific cells and tissues of *C*. *elegans*, taking advantage of the low photo-bleaching feature of LSM. The system can be adapted to image other small organisms, such as *Drosophila*, other invertebrates, as well as optically cleared mammalian tissue samples or plants including *Arabidopsis thaliana*, which can be several cm in size. Further, equipping with microfluidics platforms for easy sample handling should improve the setup to perform high-throughput light sheet microscopy of large animal groups, which would be beneficial for studying stochastic biological phenomena.

## Supporting Information

S1 Fig Measurement of light sheet thickness. All size bars correspond to 10 μm. (A) Brightfield image of a scratched glass surface. (B) and (C) show the light sheet produced on the scratched glass surface with the source of a 488nm laser and 532nm laser, respectively. (D) and (E) show a grey scale measurement over the whole field of view (dashed line in [B] and [C], respectively).
(TIF)

S2 Fig Image transformation based on landmarks. (A) Landmarks defined on the reference image A and the set Bi, which are used to align Bi images to the reference A. (B) Result of superimposing two different wavelengths (noted as red and green) without registration (left), and after registration (right).
(TIF)

S3 Fig FRAP experiment on GFP expressed specifically in *C*. *elegans* neurons. (A) Representative images of recovery at several time points after photobleaching pan-neuronal fluorescence in the posterior part of the animal. The white dashed area shows the area exposed to photobleaching, the red dashed area corresponds to ROI 2 (non-bleached area) and the green area ROI 1 shows the subset of neurons in the tail, which display fluorescent recovery. Size bars correspond to 100μm. (B) Quantification of recovering fluorescent intensity in a subset of neurons (measured in ROI1). (C) Quantification of fluorescent levels in a subset of neurons that were not photobleached.
(TIF)

S1 Table Detailed list of components required for the imaging platform. Included are vendors, description, part number, quantity and (estimate) prices. *Prices might strongly vary by the specifically requested offer. ^♯^This specific part is no longer available from this company. Prices are estimates for equivalent pieces.
(DOCX)

S1 Video Fully modeled 3D overview of the light sheet microscopy setup. Camera angles focus on: (I) the whole setup with all its compartments, (II) laser sources (green), mirror sets (grey) and shutter (yellow), (III) FRAP compartments (flip mirror and focal lens, purple), light sheet focus (orange), and imaging system (blue), (IV) cylindrical lens for production of light sheet, (V) specimen, index matching fluid bath and sample stage, (VI and VII) full overview of the whole setup.
(MP4)

S2 Video Full rotation of a 3D image of *C*. *elegans* expressing p_*myo-3*_GFP fluorescence in body wall and vulval muscles. (AVI)

S3 Video 3D movie of *C*. *elegans* expressing GFP tagged translation initiation factor eIF4E (p_*ife-2*_IFE-2::GFP) and P body reporter p_*dcap-1*_DCAP-1::dsRED fluorescence expressed in various tissues. (AVI)

S4 Video The movie sequentially shows full rotations of animal 3 from Fig 4 for three different time points (day 1, 3 and 5). (AVI)

S5 Video Sequential full 3D rotation of a small group of three animals (see Fig 5) for two time points (day 1 and day 3) upon starvation induced stress. (AVI)

S6 Video 3D movie of fluorescent recovery after photobleaching (FRAP) as happening in the anterior part of an animal expressing p_*ife-2*_IFE-2::GFP fluorescence. After each full 360° rotation a new time point of recovery is shown (see Fig 6).
(AVI)

S7 Video 3D movie of FRAP as happening in the anterior part of an animal expressing p_*dcap-1*_DCAP-1::dsRED fluorescence. After each full 360° rotation a new time point of recovery is shown (see Fig 6).
(AVI)

S8 Video 3D movie of merged data sets of S6 Video and S7 Video. (AVI)
