# Supplementary figures and images for: A Customized Light Sheet Microscope to Measure Spatio-Temporal Protein Dynamics in Small Model Organisms
Source: PLoS One. 2015 May 22;10(5):e0127869. doi: 10.1371/journal.pone.0127869 (PMC4441442; doi:10.1371/journal.pone.0127869)

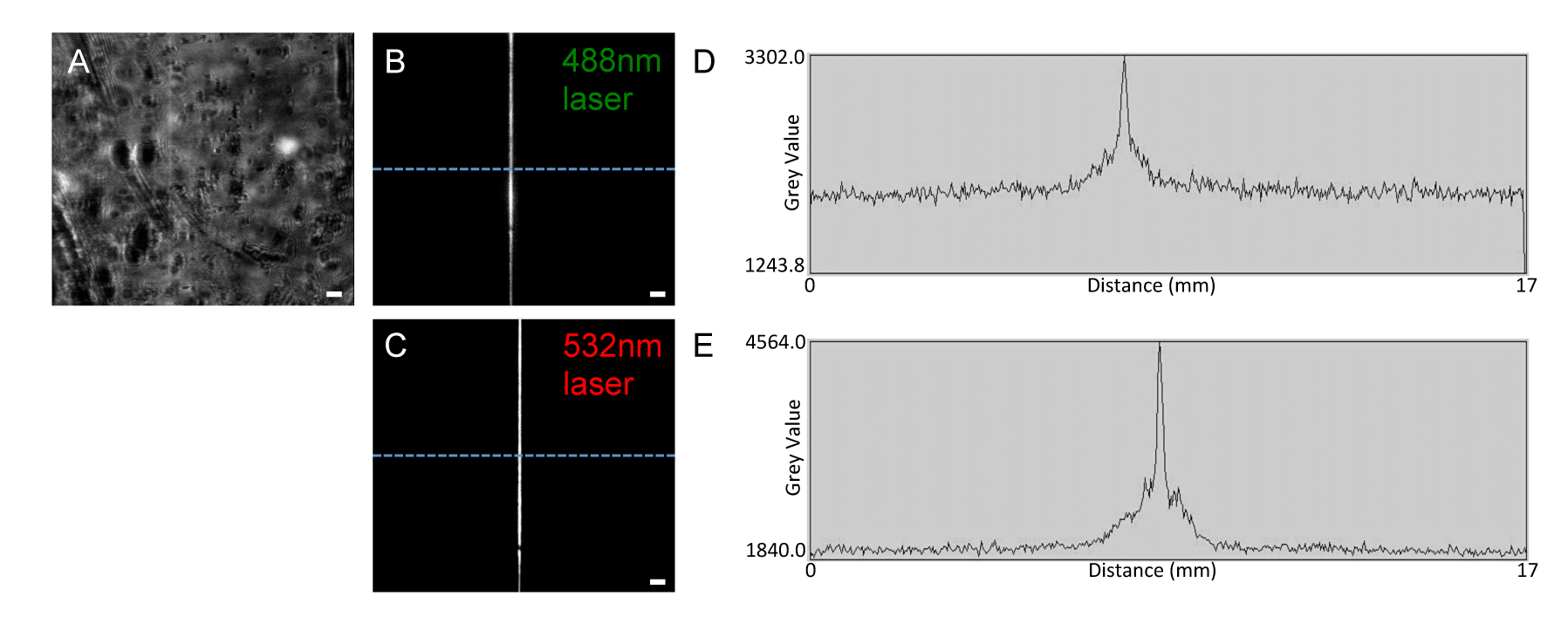

Supplement: S1 Fig — All size bars correspond to 10 μm. (A) Brightfield image of a scratched glass surface. (B) and (C) show the light sheet produced on the scratched glass surface with the source of a 488nm laser and 532nm laser, respectively. (D) and (E) show a grey scale measurement over the whole field of view (dashed line in [B] and [C], respectively). (TIF) [file pone.0127869.s001.tif]

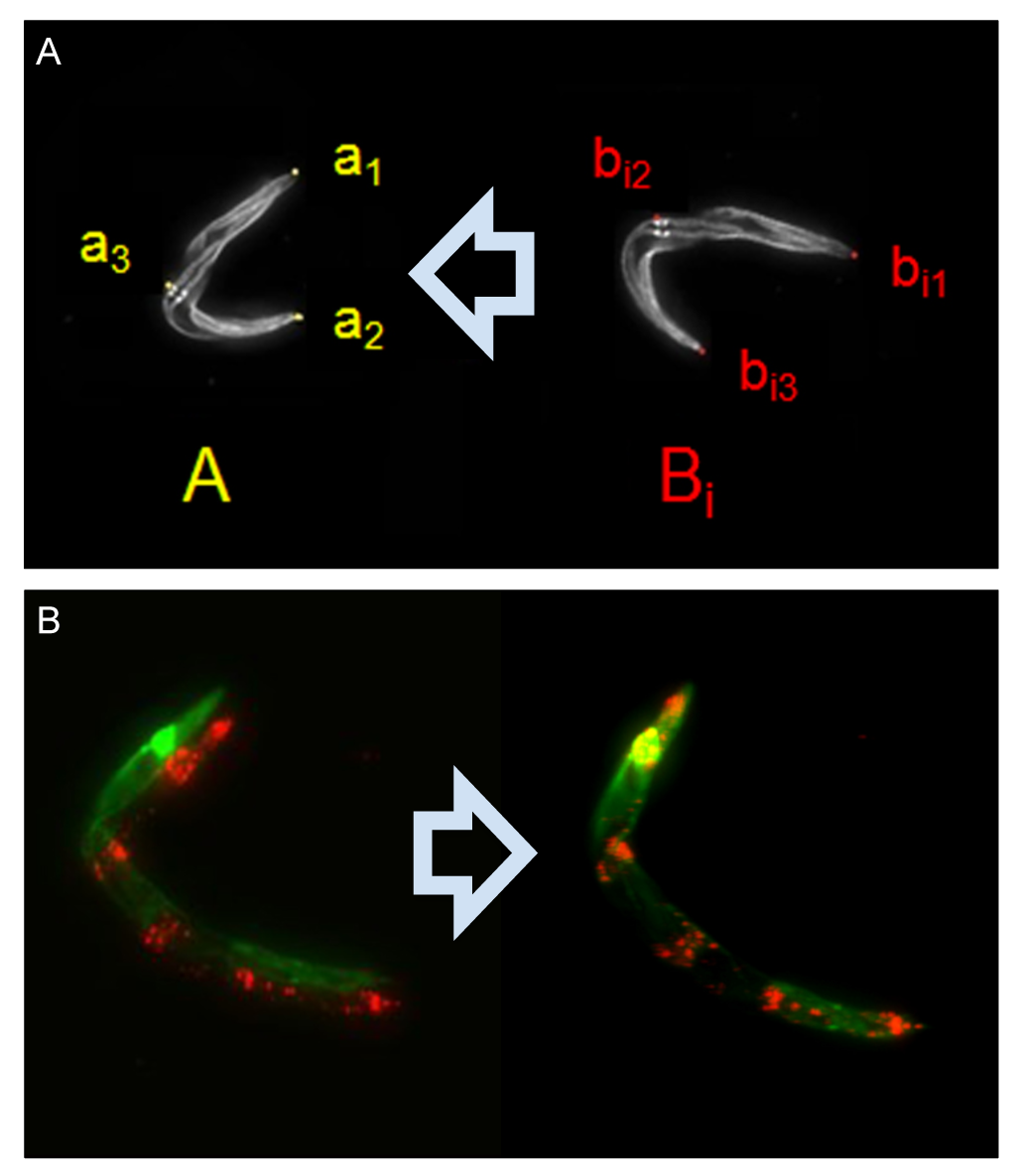

Supplement: S2 Fig — (A) Landmarks defined on the reference image A and the set Bi, which are used to align Bi images to the reference A. (B) Result of superimposing two different wavelengths (noted as red and green) without registration (left), and after registration (right). (TIF) [file pone.0127869.s002.tif]

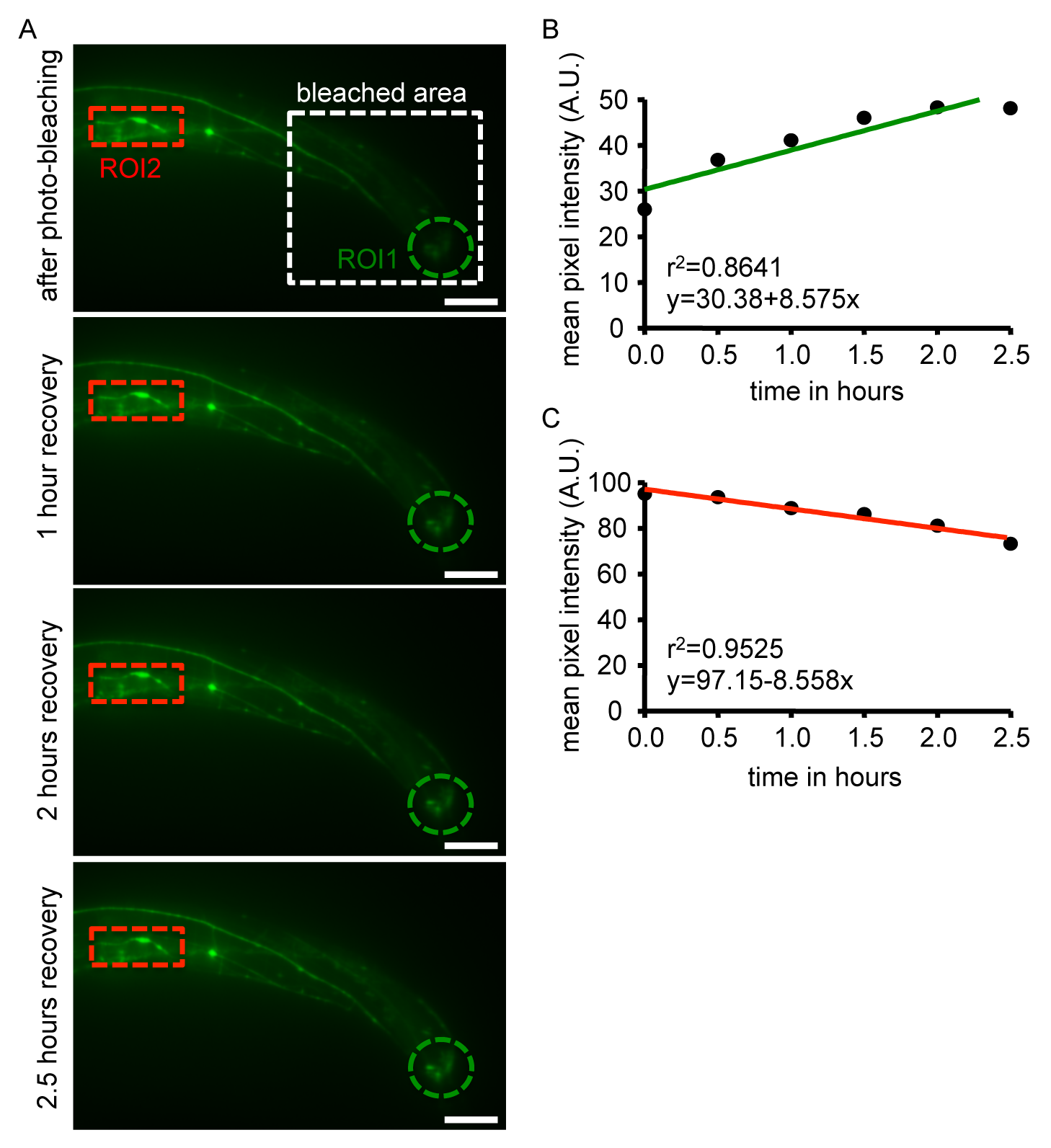

Supplement: S3 Fig — (A) Representative images of recovery at several time points after photobleaching pan-neuronal fluorescence in the posterior part of the animal. The white dashed area shows the area exposed to photobleaching, the red dashed area corresponds to ROI 2 (non-bleached area) and the green area ROI 1 shows the subset of neurons in the tail, which display fluorescent recovery. Size bars correspond to 100μm. (B) Quantification of recovering fluorescent intensity in a subset of neurons (measured in ROI1). (C) Quantification of fluorescent levels in a subset of neurons that were not photobleached. (TIF) [file pone.0127869.s003.tif]
