# Supplementary material for: A Customized Light Sheet Microscope to Measure Spatio-Temporal Protein Dynamics in Small Model Organisms
Source: PLoS One. 2015 May 22;10(5):e0127869. doi: 10.1371/journal.pone.0127869 (PMC4441442; doi:10.1371/journal.pone.0127869)
Supplement: S1 Table — Included are vendors, description, part number, quantity and (estimate) prices. *Prices might strongly vary by the specifically requested offer. ♯This specific part is no longer available from this company. Prices are estimates for equivalent pieces. (DOCX) [file pone.0127869.s004.docx]

| **Element** | **Vendor** | **Description** | **Serial Nr.** | **Quantity** | **Price in Euros** |
| --- | --- | --- | --- | --- | --- |
| **Breadboard** | Thorlabs | Optical breadboard | MB424 | 1 | 152.10 |
| **Microscope** | Edmund optics | 10x objective (infinity corrected Long WD Objective) | 46-144 | 1 | 840,75 |
|  | Edmund optics | 5x objective (infinity corrected Long WD Objective) | 46-143 | 1 | 669,75 |
|  | Edmund optics | 20x objective (infinity corrected Long WD Objective) | 46-145 | 1 | ~2000,00 |
|  | Thorlabs | Iris | SM1D12 | 1 | ~50,00 |
|  | Edmund optics | 5 cm extension tube C-Mount | 54-632 | 3 | 38,00 |
|  | Infinity | Tube lens InfiniTube™ | N/A | 1 | ~900,00^♯^ |
|  | Thorlabs | Highspeed motorized filter wheel | FW103/M | 1 | 687,49 |
|  | Standa | Motorized Rotation stage | 8MR191 | 1 | 948,00 |
|  | Standa | Motorized Translation stage | 8MT167-100 | 4 | 1045,00 |
|  | Standa | Angle bracket | 2AB167-50 | 1 | 59,00 |
|  | Custom made | Filter set | N/A |  | N/A |
| **CCD** | Andor | Ixon DV88JCS-VP | X-1639 | 1 | ~20.000,00/  On request*^♯^ |
|  | Custom made | Height spacer | N/A | 2 | N/A |
|  | Custom made | CCD-to-microscope adapter | N/A | 1 | N/A |
| **Cuvette** | Hellma | 50+/-0.5mm light path | 704.003OG | 1 | 93,70 |
|  | Custom made | Custom cuvette holder | N/A | 1 | N/A |
| **Stages/**  **Mirrors** | Edmund optics | 1.0 inch diameter kinematic mount (3-screws) | 58854 | 5 | ~60,00^♯^ |
|  | Edmund optics | Adjustable post holder  (Travel 10mm/15mm/30mm) | M6x1.0 | 3 | ~60,00^♯^ |
|  | Edmund optics | Stainless steel mounting posts (3 inch) | M4x0.7 | 1 | ~7,00 |
|  | Thorlabs | Kinematic mirror mount (2-screws) | KMS/M | 6 | 32,58 |
|  | Thorlabs | Magnetic mounting base 1’’ x 3’’ x (3/8)’’ | BA1R | 1 | 10,80 |
|  | Thorlabs | Kinematic mount with removable front plate | KS1R | 1 | 103,95 |
|  | Thorlabs | Complete periscope assembly, metric | RS99/M | 1 | 251,10 |
|  | Thorlabs | Tube lens 2’’ thread depth, one retaining ring included | SM1L20 | 1 | 14,85 |
|  | Thorlabs | Post holder base | BA1S | 1 | 4,68 |
|  | Thorlabs | f=40mm, ø1 inch achromatic doublet, SM1 threaded mount, ARC: 400-700nm | AC-254-040-A-ML | 1 | 86,31 |
|  | Thorlabs | Adjustable height collar | PSHA | 1 | 56,43 |
|  | Thorlabs | Metric ø1,5’’ mounting post bracket | C1505/M | 1 | 87,57 |
|  | Thorlabs | Aluminum breadboard 4’’ x 6’’ x (1/2)’’,  (1/4)’’-20 threaded | MB4 | 1 | 36,90 |
| **Illumination** | Hama | Telecentric backlight illuminator | LED hama MC PL M55 LINEAR | 1 | ~25,00 |
|  | Philips Lumileds | Blue LED source | N/A | 1 | N/A |
|  | Roithner | Laser source 523nm, 5.0mW | RLTMGL-523-5 | 1 | ~1.500,00* |
|  | Roithner | Laser source 488nm, 5.0mW | 473U-100  (07081601) | 1 | ~1.500,00* |
|  | Thorlabs | Mounted continuously variable ND filter ø50mm, OD: 0-4.0 | NDC-50C-4M | 1 | 393,31 |
|  | Thorlabs | He-Ne laser 594nm | N/A | 1 | ~2.000,00^♯^ |
|  | Thorlabs | 405nm blue laser module,  4.0 mW | LDM405 | 1 | 821,61 |
|  | Thorlabs | 635nm red laser module,  4.0 mW | LDM635 | 1 | 525,60 |
| **Power supply/ drivers** | VEXTA | 2-PHASE 0,9° stepping motor | PK243M-01A | 4 | ~60,00^♯^ |
|  | Roithner | Laser driver | VD-IIA DPSS | 2 | ~300,00^♯^ |
|  | Thorlabs | One channel stepper motor controller | BSC201 | 1 | 1.197,00 |
|  | Uniblitz | Single Channel Uni-stable Driver (shutter driver) | VCM-D1 | 1 | ~900,00^♯^ |
|  | Standa | USB-stepper motor controller | 8SMC1 | 2 | 505,00 |
| **Shutter** | Uniblitz | 6mm Uni-stable shutters | LS6 | 1 | ~630,00 |
